# Technology-Based Interventions for Mental Health in Tertiary Students: Systematic Review

**DOI:** 10.2196/jmir.2639

**Published:** 2013-05-27

**Authors:** Louise Farrer, Amelia Gulliver, Jade KY Chan, Philip J Batterham, Julia Reynolds, Alison Calear, Robert Tait, Kylie Bennett, Kathleen M Griffiths

**Affiliations:** ^1^Centre for Mental Health ResearchThe Australian National UniversityCanberra ACTAustralia; ^2^Young and Well Cooperative Research CentreMelbourne VICAustralia; ^3^National Drug Research InstituteCurtin UniversityPerth WAAustralia

**Keywords:** systematic review, technology, intervention, universities, students, mental health

## Abstract

**Background:**

Mental disorders are responsible for a high level of disability burden in students attending university. However, many universities have limited resources available to support student mental health. Technology-based interventions may be highly relevant to university populations. Previous reviews have targeted substance use and eating disorders in tertiary students. However, the effectiveness of technology-based interventions for other mental disorders and related issues has not been reviewed.

**Objective:**

To systematically review published randomized trials of technology-based interventions evaluated in a university setting for disorders other than substance use and eating disorders.

**Methods:**

The PubMed, PsycInfo, and Cochrane Central Register of Controlled Trials databases were searched using keywords, phrases, and MeSH terms. Retrieved abstracts (n=1618) were double screened and coded. Included studies met the following criteria: (1) the study was a randomized trial or a randomized controlled trial, (2) the sample was composed of students attending a tertiary institution, (3) the intervention was delivered by or accessed using a technological device or process, (4) the age range of the sample was between 18 and 25 years, and (5) the intervention was designed to improve, reduce, or change symptoms relating to a mental disorder.

**Results:**

A total of 27 studies met inclusion criteria for the present review. Most of the studies (24/27, 89%) employed interventions targeting anxiety symptoms or disorders or stress, although almost one-third (7/24, 29%) targeted both depression and anxiety. There were a total of 51 technology-based interventions employed across the 27 studies. Overall, approximately half (24/51, 47%) were associated with at least 1 significant positive outcome compared with the control at postintervention. However, 29% (15/51) failed to find a significant effect. Effect sizes were calculated for the 18 of 51 interventions that provided sufficient data. Median effect size was 0.54 (range –0.07 to 3.04) for 8 interventions targeting depression and anxiety symptoms and 0.84 (range –0.07 to 2.66) for 10 interventions targeting anxiety symptoms and disorders. Internet-based technology (typically involving cognitive behavioral therapy) was the most commonly employed medium, being employed in 16 of 27 studies and approximately half of the 51 technology-based interventions (25/51, 49%). Distal and universal preventive interventions were the most common type of intervention. Some methodological problems were evident in the studies, with randomization methods either inadequate or inadequately described, few studies specifying a primary outcome, and most of the studies failing to undertake or report appropriate intent-to-treat analyses.

**Conclusions:**

The findings of this review indicate that although technological interventions targeting certain mental health and related problems offer promise for students in university settings, more high quality trials that fully report randomization methods, outcome data, and data analysis methods are needed.

## Introduction

University students are predominantly at an age when the incidence of mood and anxiety disorders is peaking—mental health problems are most likely to begin before 24 years of age [1]. Combined with the stresses that the transition to university may provoke [2], it is not surprising that mental disorders are responsible for a high level of disability burden in university students [3-5], with a recent US study reporting a prevalence level of nearly 50% in the previous year [6]. Mental disorders also have a negative impact on academic participation and outcomes [1,3,7]. Universities provide a unique opportunity to prevent and treat mental disorders in a high-risk group and have the potential to host comprehensive approaches that cover prevention, early intervention, and treatment strategies [1,3,8]. However, many universities have limited resources available to support comprehensive approaches to student mental health, and students are often reluctant to seek help from traditionally structured student counseling centers [5,8]. It is estimated that less than one-quarter of university students with a mental disorder seek help during any 1 year [6]. Consequently, only a minority of university students with mental health problems receive adequate help [4].

Electronic media has the potential to play a significant role in developing university-based approaches to improving mental health. It is reported that young people seek help or information for emotional and mental health problems online [9-11] and, in the university population, electronic media seems to be useful in screening, increasing mental health literacy, and encouraging at-risk students to access support and treatment [1,5,8,12,13]. Online interventions may be highly relevant to university populations as they can be easily accessed, are cost effective for large populations, and may be perceived as less stigmatizing than traditional approaches to care [14,15]. Universities have traditionally delivered mental health services in clinical settings, such as face-to-face individual or group-based consultations [8], which tend to be more costly and time-intensive than distal interventions [15]. Consequently, there is a need to identify effective mental health interventions that can be distributed to students in a virtual setting and cover the spectrum of interventions from prevention to treatment.

Numerous studies and several reviews have evaluated Internet-based and non-Internet-based interventions for substance misuse and eating disorders in tertiary student populations [16-22]. However, relatively few studies have focused on the online interventions for mood disorders, anxiety disorders, or other mental health issues in university students. One review has examined the effectiveness of anxiety and depression interventions of any modality in a higher education setting [1]. Most of the identified studies adopted a face-to-face approach and were time consuming and costly. However, the authors suggested that the Web may be an ideal way to deliver promising interventions to higher education students [1].

General population reviews of Web-based depression and anxiety interventions have indicated that such interventions can be effective for treating common mental disorders, with moderate to large effect sizes [23-25]. However, with the exception of eating disorders and substance misuse, there have been no systematic reviews specifically targeted at the effectiveness of Internet or other technology-based mental health interventions for university students. Consequently, the present study comprises a systematic review of published randomized trials of technology-based interventions which included a mental health symptoms outcome measurement and were evaluated in a university setting for disorders other than substance use disorders and eating disorders. The aim of this review is to evaluate both the effectiveness of these interventions and the methodological quality of studies identified in the systematic literature search.

## Method

### Search Methodology

The PubMed, PsycInfo, and Cochrane Central Register of Controlled Trials databases were searched using keywords, phrases, and Medical Subject Headings (MeSH) terms in May 2012. The search strategy (see Multimedia Appendix 1) involved terms that covered 3 broad concepts: (1) setting or population in which the intervention was conducted (university) AND (2) the focus of the intervention (mental disorder/mental health promotion), AND (3) the modality in which the intervention was delivered or accessed (technology such as the Internet, telephone, etc). Keywords, MeSH terms, and phrases pertaining to concept 1 (university) and concept 3 (technology) were developed by the researchers. Those pertaining to concept 2 (mental disorder/health) were derived from the International Classification of Diseases (ICD-10) list of mental disorders from the National Health and Medical Research Council (NHMRC) keywords for mental health research and additional terms identified by the researchers. The present review conforms to the Preferred Reporting Items for Systematic Reviews and Meta-Analyses (PRISMA) statement [26]. A PRISMA checklist is provided in Multimedia Appendix 2.

### Study Identification

**Figure 1:**
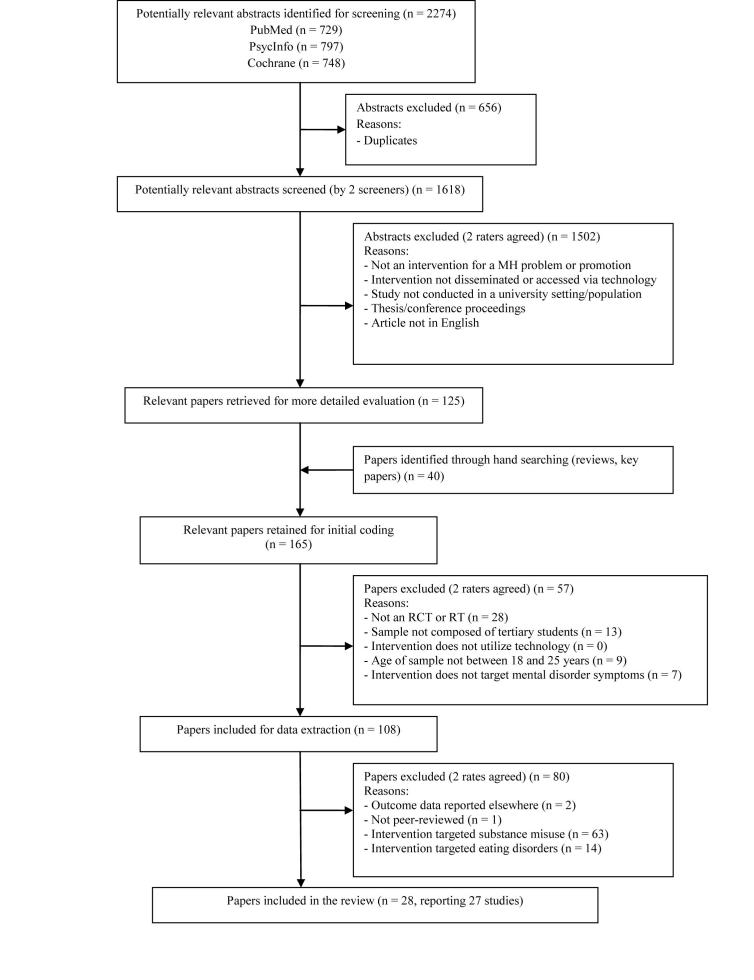
Study identification flow diagram.

Figure 1 displays the flowchart for the selection of the included studies, which involved multiple stages. The first stage involved screening to eliminate clearly irrelevant abstracts. A total of 2274 abstracts were returned by the database searches, of which 656 abstracts were identified as duplicates and excluded. The remaining 1618 abstracts were screened by 2 raters (LF or AG and JC) according to the following criteria:

1. The study investigated (1) an intervention for a mental health problem or disorder, or the promotion of positive mental health, or (2) the study measured a mental health–related outcome in relation to the intervention.

2. The intervention was either disseminated via or accessed using a technological device (eg, computer, smartphone, telephone) or process (eg, email, Internet, SMS/text-messaging, video).

3. The study was conducted in a university setting with students or young people.

4. The study was not a thesis or a conference proceeding.

5. The article was written in English.

Studies that were considered relevant by both raters were retained and those that were identified as relevant by only 1 rater were rescreened by both raters according to the preceding criteria. Following the second screen, abstracts that both raters considered relevant were retained. The remaining abstracts were discussed by the 2 raters and relevant abstracts were mutually agreed upon following discussion. A total of 125 abstracts were identified as relevant following the initial screening stage. An additional 40 papers were located through handsearching the reference lists of papers from the initial 125 identified abstracts and reviews located through the original 1618 abstracts. In addition, JC screened the reference lists on Beacon [27], a portal listing online applications for mental and physical disorders. This yielded a total of 165 papers that underwent the second stage of screening and were included if they met the following stricter criteria:

1. Study design: the study was a randomized controlled trial (RCT) or a randomized trial (ie, an equivalence trial).

2. Recruitment population: the sample was composed of students attending a tertiary institution, such as university, college, or a Technical and Further Education (TAFE) institution.

3. Intervention type: the intervention or some portion of the intervention (eg, reminder or follow-up contact) was either delivered by or accessed using a technological device or process (Internet, telephone, video). Studies that used technology only to conduct screening or measure outcomes (which are not considered part of the intervention) did not satisfy this criterion.

4. Age: the age range of the sample was between 18 and 25 years or the mean age of the sample was between 18 and 25 years. If sample age was not able to be sourced directly from the authors, studies that sampled undergraduates without specifying age were included.

5. Intervention focus: the intervention was designed to improve, reduce, or change symptoms relating to a mental disorder (as defined by the DSM-IV and ICD-10).

Studies that were considered relevant by both raters were retained and those that were identified as relevant by only 1 rater were rescreened by a third rater. A total of 108 papers were retained for coding by 2 coders (LF or AG and JC). Three of these papers [28-30] were subsequently excluded for the following reasons: outcome data reported elsewhere (ie, summary papers, n=2) [28,30], and a non-peer-reviewed conference proceeding (n=1) [29]. Of the remaining 105 papers, 63 papers examined interventions for substance use problems (alcohol, tobacco, and other drugs) and 14 papers examined interventions for eating disorders, weight gain, or body image. These papers were excluded from the current review as they have been the subject of previous reviews. The remaining 28 papers included in this review examined interventions for other mental health problems and related issues (depression, anxiety disorders, stress, Internet addiction, psychological distress, and hardiness/acculturation).

### Coding of the Included Papers

A total of 28 papers were included. However, 2 papers [31,32] reported data from the same study leaving a total of 27 studies for analysis. Each of the 27 included studies was coded by 2 raters (LF or AG and JC) with a preformulated rating sheet with relevant data extracted and recorded. Data coding comprised the following: country where the study was conducted, participant characteristics and recruitment method, type and length of intervention and the technology used, whether or not the intervention was distal, amount of human contact involved in the intervention, whether or not intention-to-treat (ITT) analysis was employed, an overall quality rating for the study, the primary outcome measure for the study, measurement occasions, whether or not the intervention was significantly superior to the control at each measurement occasion, and Hedge’s *g* effect sizes for the difference between each intervention and the control group at each measurement occasion.

Type of intervention (ie, intervention target group) was categorized using the framework described by Mrazek and Haggerty [33]. *Universal programs* were those available for all (no screening involved), *selective programs* were those that selected individuals at risk of a mental health condition (involved screening), *indicated programs* were those that selected individuals displaying symptoms of a mental health condition in the absence of a mental disorder (involved screening), and *treatment programs* were those that targeted individuals diagnosed with a mental disorder.

Amount of human contact was coded based on categories identified by Newman and colleagues [34]: (1) self-administered therapy (therapist/human contact for assessment, at most), (2) predominantly self-help (therapist/human contact beyond assessment for periodic check-ins, teaching clients how to use the self-help tool, and/or for providing the initial therapeutic rationale; if any assistance in the use of therapeutic tools was provided, it did not involve more than 1.5 hours of the therapist’s/human’s time), (3) minimal-contact therapy (active involvement of a therapist/human, although to a lesser degree than traditional therapy for this disorder; including any treatment in which the therapist/human assisted the client in the application of specific therapy techniques and that involved more than 1.5 hours of the therapist’s/human’s time), and (4) predominantly therapist-administered treatments (clients had regular contact with a therapist/human for a typical number of sessions, but the study attempted to determine whether the use of a self-help tool augmented the impact of the standard therapy). In studies in which reminders were provided and no human involvement or tailoring was reported, the reminders were considered to be automated, and the study was categorized as self-administered. Interventions were considered to be distal if they “traveled” to the recipient, rather than the recipient being required to physically go to a location to participate in the intervention.

Study quality was assessed using the risk of bias criteria proposed by the Cochrane Effective Practice and Organisation of Care Group [35], a measure designed to assess potential sources of bias for studies involving a control group. Items are designed to measure bias relating to inadequate random allocation sequence and allocation concealment, differences in baseline outcome measurements and characteristics, inadequate treatment of missing outcome data, researcher knowledge of allocated interventions, contamination between the conditions, selective outcome reporting, as well as any other risk of bias. A score of 1 was awarded for each criterion adequately addressed within the paper with potential scores ranging from 0 to 9.

### Data Analysis

A quantitative meta-analysis was not conducted because of the heterogeneous nature of the studies. Descriptive information regarding whether the study reported a significant time × group interaction was reported. This information was reported for the primary symptom outcome measure(s) as specified by the authors. In the event that a primary outcome was not specified or multiple measures of the same construct were examined (eg, multiple measures of depressive symptoms), only the first outcome that was described in the measures section of the paper was reported. Where possible, Hedge’s *g* effect sizes were calculated using mean posttest and follow-up scores and standard deviations for each intervention group and the control group. Effect sizes were not calculated in several instances in which means and standard deviations were not reported at all or were not reported for the overall sample, or if the number of participants analyzed in the intervention and control groups was unclear or not reported. Negative effect sizes indicate that the control group outperformed the intervention group. For studies that conducted both ITT and completer analyses, results pertaining to the ITT analyses were reported. Analyses for follow-up were not conducted because of the heterogeneity of the follow-up periods. The association between various study characteristics and whether or not studies reported statistically significant results at postintervention favoring the intervention was explored using a series of Fisher exact tests for categorical variables and Mann-Whitney *U* tests for continuous variables. Data were analyzed by comparing intervention and control conditions within studies. For completeness, all interventions are presented in Multimedia Appendix 3 (4 interventions did not employ technology). However, only interventions that contained technology were compared for analysis. Therefore, from the 27 studies, a total of 51 comparisons were made (each study could examine more than 1 comparison).

## Results

### Study Characteristics

Multimedia Appendix 3 shows the characteristics of the included studies (N=27). Studies were categorized according to the symptoms or disorder that the intervention targeted as reported by the study authors. The conditions targeted were depression and anxiety (n=7) [36-42], anxiety symptoms (n=4) [31,43-45], examination anxiety (n=4) [46-49], specific phobia (n=3) [50-52], stress (n=2) [53,54], social anxiety (n=1) [55], computer-related anxiety (n=1) [56], posttraumatic stress (n=1) [57], generalized anxiety disorder (n=1) [58], psychological distress (n=1) [59], hardiness and acculturation (n=1) [60], and Internet addiction (n=1) [61].

### Origin

Most studies targeting depression and anxiety were conducted in the United States [36-38,40,41] with the remaining 2 from Australia [39,42]. Studies targeting anxiety disorders and stress were conducted in a wider range of countries with 7 from the United States [44,46,50,51,53,54,58], 4 from Italy [43,45,47,49], and 1 each from Australia [31], United Kingdom [48], Belgium [52], Spain [55], and the Netherlands [57]. Studies targeting other mental health issues were from the United States [60], United Kingdom [59], and China [61].

### Interventions

#### Intervention Type

Ten studies employed universal interventions, and fewer studies focused on indicated (n=7), selective (n=7), and treatment (n=3) interventions.

#### Depression and Anxiety Symptoms (n=7)

Of the studies for depression and anxiety symptoms, 43% (3/7) examined selective interventions [39,41,42], with the remaining studies assessing universal (2/7, 29%) [36,37], indicated (1/7, 14%) [38], and treatment (1/7, 14%) [40] interventions. The 4 selective or indicated studies [38,39,41,42] evaluated cognitive behavioral therapy (CBT)-based interventions, the 2 universal intervention studies [36-37] focused on relationship skills training, and the treatment study [40] examined the effectiveness of an intervention based on physical activity and Web-based social cognitive theory (SCT) as an adjunct to mental health counseling.

#### Anxiety Symptoms (n=4)

Of the 4 studies targeting anxiety symptoms, 3 were universal [44,45] and 1 was indicated [31]. The 3 universal studies delivered either relaxation [43,45] or exposure-based interventions [44], and the indicated study delivered CBT [31].

#### Examination Anxiety (n=4)

Of the 4 studies targeting examination anxiety, 2 were universal [47,49], 1 was selective [48], and 1 was indicated [46]. The 2 universal studies delivered stress inoculation training [47,49], the selective study delivered CBT or education interventions [48], and the indicated study delivered systematic desensitization and relaxation [46].

#### Specific Phobia (n=3)

Of the 3 studies targeting specific phobias, 2 were indicated studies [50,51] and 1 was a treatment study [52]. The 2 indicated studies targeted spider phobia [50] and acrophobia [51], and delivered exposure therapy. The treatment study targeted spider phobia and delivered exposure therapy [52].

#### Stress (n=2)

Of the 2 studies targeting stress, 1 was universal [54] and 1 was selective [53]. The universal study delivered real and virtual reality physical activity [54] and the selective study delivered health information and motivational feedback interventions [53].

#### Other Anxiety Disorders (n=4)

The study targeting computer-related anxiety was universal and delivered hypnosis or biofeedback [56]. The study targeting posttraumatic stress was selective and delivered structured writing [57]. The study targeting generalized anxiety disorder was indicated and delivered exposure, expressive writing, or auto-photic stimulation (APS) interventions [58]. The study targeting social anxiety disorder was a treatment study delivering CBT [55].

#### Other Mental Health Problems and Issues (n=3)

For the 3 studies examining other issues, 1 used a universal intervention targeting hardiness and acculturation [60], 1 was an indicated study focused on Internet addiction [61], and 1 was a selective study targeting psychological distress [59]. Two of the studies delivered education [59,60], and the remaining study delivered motivational interviewing [61].

### Technology Employed

The 51 interventions examined in the present review employed a range of broad technology types including the Internet (n=18), audio (n=9), virtual reality (n=6), video (n=4), stand-alone computer programs (n=1), and/or a combination of these (Internet plus computer program, n=5; audio plus video, n=5; computer plus audio, n=1; Internet plus audio, n=1; Internet plus APS, n=1). There were no telephone-only interventions. The interventions were delivered using a range of specific devices, including computer (n=24), mobile phone (n=4), Moving Picture Experts Group Layer-3 (MP3) audio file (n=3), Digital Versatile Disc (DVD; n=3), compact disc (CD; n=2), virtual reality devices (n=6), audiotape player (n=4), video player (n=2), and combinations of these, including computer plus audio player (n=2) and computer plus APS (n=1). CBT interventions tended to be Internet-based and were commonly delivered using websites and in conjunction with therapist support in-person or via email. Email was the most common method of monitoring. Educational interventions tended to be delivered using stand-alone computer-based programs and videos. Interventions involving exposure, stress inoculation training, and relaxation tended to be delivered via audio (audiotape, CD, and MP3), combined audio and video (DVD), mobile phone, or virtual reality.

### Intervention Length and Delivery

Intervention length ranged from 15 minutes to 10 weeks. For interventions of less than 1 week in duration, intervention length ranged from 15 to 60 minutes (mean 34.23, SD 13.82). For interventions that were 1 week or longer, the mean intervention length was 4.1 weeks (SD 3.04). Length of time to follow-up ranged from immediately postintervention to 12 months postintervention. Of the 51 technology-based interventions employed, 27 (52.9%) were delivered distally, 18 (35.3%) were delivered nondistally, and 6 (11.8%) contained distal and nondistal components. Of the 25 Internet-based interventions, 13 (52.0%) were completely distal, 6 (24.0%) contained a combined distal and nondistal component, and 6 (24.0%) interventions were not distal [39,42,61].

### Level of Human Contact

Over half of the interventions were self-administered (30/51, 59%), and approximately one-fifth were predominantly self-help (10/51, 20%). For interventions that were predominantly self-help, human contact was most commonly provided in the form of email monitoring or moderation of a discussion forum. Four interventions (8%) involved minimal contact and tended to include more intensive therapist involvement via email. Interventions classified as therapist administered (7/51, 14%) were often face-to-face interventions that served as comparison groups to a technology-based intervention, or were face-to-face interventions with a technology-based component as an adjunct (ie, Internet-based homework) [40,42].

### Participants

By definition, the mean age of participants fell between 18 and 25 years. Most samples were composed solely of undergraduate university students. Two studies targeted specific groups of students: nursing students [44] and Asian-Indian students [60]. Females formed the majority of participants in most studies, with the exception of 5 studies in which males were either the majority (n=3) [46,51,60] or the sample contained equal numbers of males and females (n=2) [43,45]. The most common recruitment methods were university-wide emails, advertisements in university publications or during lectures, and flyers posted around university campuses. In 7 studies, samples were recruited through undergraduate psychology or health courses [31,36,38,39,50,54,56].

### Outcome Measures

Four of the 7 depression and anxiety studies used the Beck Depression Inventory (BDI) and the Beck Anxiety Inventory (BAI) as their primary outcome measures [36-38,41]. Three of the 4 studies targeting anxiety symptoms used the State Trait Anxiety Inventory (STAI) as their primary outcome measure [43-45], as did 2 of the 4 studies targeting examination anxiety [47,49]. The 2 remaining studies targeting examination anxiety used the Test Anxiety Inventory (TAI) [46,48]. Studies targeting specific phobias used the Fear of Spiders Questionnaire [50], the Acrophobia Questionnaire [51], and an 11-point fear rating scale [52]. The 2 studies targeting stress used either the Perceived Stress Scale [53] or the Momentary Mood States Checklist [54]. The single studies targeting social anxiety, computer-related anxiety, posttraumatic stress, generalized anxiety disorder, psychological distress, hardiness, acculturation and social support, and Internet addiction used measures specific to the disorder being targeted.

### Study Quality

Sample sizes across all studies ranged from 20 to 283 (median 60). Most studies were RCTs (n=26), and 1 study was a randomized trial [46]. Of the 26 RCTs, 10 studies employed a no-intervention control, 9 studies used attention control groups, 6 studies used a wait-list control, and in 1 study, participants were assigned to a wait-list control but also received treatment as usual. Quality ratings for the studies employing a control group ranged from 1 to 6, with an overall mean rating of 4.42 of a possible 9 points. The mean quality ratings for categories of studies were stress (mean 5.0, range 4-6); other anxiety disorders, such as seasonal affective disorder, posttraumatic stress disorder, generalized anxiety disorder, and computer-related anxiety (mean 5.0, range 4-6); other issues, such as psychological distress, acculturation, and Internet addiction (mean 4.67, range 3-6); depression and anxiety symptoms (mean 4.43, range 3-6); specific phobias (mean 4.33, range 3-5); anxiety symptoms (mean 4.0, range 3-5); and examination anxiety (mean 3.67, range 1-6). Table 1 shows the number of studies that satisfied each of the quality rating criteria.

**Table 1:** Numbers (and percentages) of studies (with control groups) meeting quality rating criteria of the Cochrane Effective Practice and Organisation of Care (EPOC).

Criterion #	EPOC quality rating criteria	Studies, n (%)
1	Was the allocation sequence adequately generated?	5 (19.2)
2	Was the allocation adequately concealed?	1 (3.8)
3	Were baseline outcome measurements similar?	17 (65.4)
4	Were baseline characteristics similar?	11 (42.3)
5	Were incomplete outcome data adequately addressed?	10 (38.5)
6	Was knowledge of the allocated interventions adequately prevented during the study?	0 (0)
7	Was the study adequately protected against contamination?	24 (92.3)
8	Was the study free from selective outcome reporting?	21 (80.8)
9	Was the study free from other risks of bias?	26 (100)

As indicated in Table 1, few studies used or reported adequate randomization methods. In terms of baseline outcome measurement, more than half of studies reported that there were no significant differences present across study groups at baseline. Less than half of studies, however, reported that the characteristics of the providers of the intervention and control conditions were similar (criteria 4). Approximately one-third of studies reported that they used methods to adequately address incomplete data. No studies met criteria 6 (Was knowledge of the allocated interventions adequately prevented during the study?).

Of the entire 27 studies, 8 studies undertook ITT analyses, and 13 did not. Six studies did not report this information. Of the 8 ITT studies, half (n=4) reported data from a full sample (no attrition), 2 used maximum likelihood estimation methodology, 1 used the last observation carried forward, and 1 used a mixed models analysis.

### Intervention Efficacy

#### Depression and Anxiety Symptoms

Among the 7 studies targeting depression and anxiety symptoms, there were 10 interventions that were compared to a control group (some studies had multiple intervention arms). Six interventions were CBT-based (delivered either online or using a stand-alone computer), 2 interventions involved relationship focused skills training, 1 intervention comprised physical activity and SCT, and 1 intervention involved online peer-support.

##### Effective/Mixed Results

Postintervention, 3 of the CBT-based interventions were associated with a significant time × group interaction favoring the intervention group on both depression and anxiety symptom outcomes. The remaining CBT interventions (n=3) only found effects for anxiety symptoms postintervention, as did the online peer-support intervention. Only 1 of the relationship skills training interventions found a significant interaction at posttest for depression symptoms [36]. The second relationship skills training intervention study found a positive effect for anxiety at 10-month follow-up [37].

##### Not Effective

The physical activity and Web-based SCT intervention did not find a significant group × time interaction postintervention for either depression or anxiety [40].

#### Anxiety Symptoms

Among the 4 studies targeting anxiety symptoms, 9 interventions were examined. Six interventions were relaxation-based: video plus an audio narrative (n=2), video alone (n=1), audio narrative alone (n=2), and virtual reality headset plus audio narrative (n=1). Two interventions were exposure-based: audiotape alone (n=1) and audiotape plus progressive muscle relaxation (n=1). One intervention was CBT-based.

##### Effective/Mixed Results

The 2 exposure-based interventions were effective for reducing anxiety relative to a control condition [44]. Video and audio relaxation combined was associated with significant within-group decline in anxiety symptoms in 1 study [43], but data was not compared with a control group and this intervention was also not found to be effective in another study [45].

##### Not Effective

Video alone, audio alone, and a virtual reality headset plus an audio narrative were not found to be effective for reducing anxiety symptoms [43,45]. The only online CBT intervention was also not associated with a significant interaction in favor of the intervention [31].

#### Examination Anxiety

Among the 4 studies targeting examination anxiety, 11 interventions were examined. Two interventions examined computer-assisted exposure plus audio relaxation, 8 interventions examined stress inoculation delivered by video and audio (n=3), video alone (n=2), and audio alone (n=3), and 1 intervention examined online CBT.

##### Effective

One study examining 4 stress inoculation interventions (video plus audio vs video only vs audio via MP3 only vs audio via CD only) found that all interventions were effective in reducing anxiety symptoms relative to a no-intervention control condition [47]. Online CBT was also found to be effective for symptoms of examination anxiety [48]. The study examining exposure plus audio relaxation found that computer-based delivery was equivalent to group-based delivery of the intervention [46].

##### Not Effective

The remaining study examining stress inoculation interventions did not provide sufficient data to determine the effectiveness of the interventions relative to the control group [49].

#### Specific Phobia

Among the 3 studies targeting specific phobia, 5 interventions were examined. All interventions were exposure-based. Three were delivered using virtual reality and 2 were delivered using video.

##### Effective

Virtual reality exposure interventions for spider phobia [50] and acrophobia [51] were associated with significant reductions in anxiety symptoms relative to a control group. Exposure using video was also effective in the treatment of spider phobia [52].

#### Stress

Among the 2 studies examining stress, 4 interventions were examined. Interventions included online education (n=1) or online motivational feedback (n=1) [53], a virtual reality simulation of the outdoors while walking on a treadmill (n=1), or a virtual reality simulation alone (n=1) [54].

##### Not Effective

None of the interventions were effective in reducing stress.

#### Other Anxiety Disorders

The study targeting social anxiety disorder examined online CBT [55]. The study targeting computer-related anxiety examined computer-assisted biofeedback [56]. The study targeting posttraumatic stress examined online structured writing exercises [57]. The study targeting generalized anxiety disorder examined 3 interventions: online exposure, online expressive writing, and APS [58].

##### Effective

Postintervention, online CBT was found to be effective for treating social anxiety disorder [55], biofeedback was effective for symptoms of computer-related anxiety [56], structured writing was effective for symptoms of posttraumatic stress [57], and online exposure and APS were effective for symptoms of generalized anxiety disorder [58].

##### Not Effective

Online expressive writing was not found to be effective for symptoms of generalized anxiety disorder [58].

#### Other Mental Health Problems and Issues

One study targeting psychological distress examined 2 interventions: online education and online education plus an online support group [59]. One study targeting hardiness, acculturation, and social support examined online information [60]. One study targeting Internet addiction examined 3 interventions: online motivational interviewing with feedback in a laboratory setting, online motivational interviewing without feedback in a laboratory setting, and online motivational interviewing without feedback in the participant’s own setting [61].

##### Effective/Mixed Results

An online education intervention and a social support intervention each demonstrated within-group decline in psychological distress over time, but were not compared with a control group [59]. However, a combined intervention involving both the online education intervention and the support group was not more effective than education alone [59]. All of the motivational interviewing interventions targeting Internet addiction were associated with significant within-group decline in symptoms over time [61], but the interaction effect with the control was not tested. However, the control group did not show significant within-group decline over time.

##### Not Effective

Online information was not found to be effective in the study targeting hardiness and acculturation [60].

### Effect Sizes

For interventions targeting depression and anxiety symptoms with available data (n=8), effect sizes ranged from –0.07 to 3.04 (median 0.54; depression = 0.48, anxiety = 0.77). Across interventions targeting anxiety symptoms and disorders with available data (n=10), effect sizes ranged from 0.07 to 2.66 (median 0.84). Because of insufficient or unavailable data, effect sizes were unable to be calculated for 33 of the 51 interventions (64%) or 14 of the 27 studies (52%), which included all of the interventions targeting stress, computer anxiety, psychological distress, hardiness and acculturation, and Internet addiction.

Less than half of studies provided sufficient data to calculate effect sizes. For interventions that targeted depression and anxiety, effect sizes were as follows for the 1 universal (alpha = –0.74), 6 selective (alpha = 0.81), 1 indicated (alpha = 0.54), and 1 treatment (alpha = 0.18) interventions. For interventions targeting anxiety symptoms and disorders, none of the 16 universal interventions (5 trials) had had sufficient data to calculate effect size. Alpha levels were as follows for the 3 selective interventions (alpha = 0.67), 5 indicated interventions (alpha = 0.49), and 2 treatment interventions (alpha = 1.83).

### Association Between Positive Outcomes and Study Characteristics

Mann-Whitney *U* tests demonstrated no association between study outcome favoring the intervention and the following study characteristics: number of intervention sessions (*U*=35.0, *P*=.21), length of intervention (weeks; *U*=43.5, *P*=.67), sample size (*U*=40.5, *P*=.53), and quality rating (*U*=41.5, *P*=.56). Fisher exact tests also demonstrated no association between study outcome favoring the intervention and type of control group (attention placebo: n=9; wait list: n=6; no intervention: n=10, *P*=.84); type of technology used (Internet, includes all interventions that are Internet-based or involved an Internet component: n=34; other: n=21, *P*=.57); whether or not the intervention was distal (yes, includes completely and partially distal interventions: n=33; no: n=22; *P*=.74); and amount of human contact (self-administered: n=30; predominantly self-help: n=10; minimal-contact therapy: n=4; predominantly therapist-administered treatments: n=7; *P*=.30). The success rates of different types of interventions at achieving a study outcome favoring the intervention appeared dissimilar between the universal (n=25, 56%), selective (n=12, 67%), indicated (n=13, 75%), and treatment (n=5, 80%) trials. However, chi-square tests demonstrated that this difference was not significant (*P*=.74).

## Discussion

### Principal Findings

This systematic review identified 27 studies reporting RCTs of technology interventions targeting depression, anxiety, and related mental health issues, excluding substance misuse and eating disorders. Most of the studies (24/27, 89%) employed interventions focused on anxiety symptoms or disorders or stress, although 29% of these 24 studies (n=7) targeted both depression and anxiety. No study reported that they targeted depression alone in this population. Internet-based technology (typically involving CBT) was the most commonly employed medium, and it was used in 16 studies and almost half of the interventions. Distal and universal preventive interventions were the most common type of intervention. No study investigated the effectiveness of telephone interventions, and only 3 of 27 interventions (11%) targeted treatment. Audio and video were commonly used for exposure, stress inoculation, and relaxation training. More trials were undertaken in the United States than in any other country (13/27, 48%).

Overall, approximately half (n=24, 47%) of the 51 technology interventions were associated with at least 1 significant positive outcome compared with the control at postintervention, with 29% (n=15) failing to find a significant effect. Only 2 interventions (from 1 study) did not have a control group for comparison, and the remaining 10 interventions (from 3 studies) did not provide sufficient data on interaction effects to determine efficacy compared with the control. The studies finding a positive outcome compared with the control included 7 of the 10 technology-related interventions employed in the anxiety and depression studies, 2 of the 9 technology interventions in the anxiety studies, all 5 of the specific phobia interventions, none of the 4 stress interventions, and 4 of 5 interventions targeting other anxiety disorders. None of the 6 interventions targeting other conditions were demonstrated to be effective relative to controls, with 3 of these belonging to 1 study that did not provide sufficient data to determine efficacy compared with control [61]. Similarly, mixed results have been found in prior reviews of both Internet-based eating disorder prevention interventions [20] and in Internet-based alcohol use interventions [62].

Thus, the findings of the current review indicate that technological interventions targeting certain mental health and related problems offer promise for students in university settings. The data suggest that technology-based CBT may be particularly useful in targeting anxiety and, to a lesser extent, depressive symptoms in interventions targeting both depression and anxiety. A previous review on Internet-based interventions found comparable effect sizes for both depression (alpha = 0.42 to 0.65) and anxiety (alpha = 0.29 to 1.74) preventive and treatment interventions involving CBT [24]. Exposure approaches, including those involving virtual reality technology, offer promise for specific phobias. Moreover, the focus on universal intervention and prevention approaches including selective and indicated samples and the evidence that these may be effective are promising, especially given findings in the general community that optimal and comprehensive current treatment approaches alone cannot avert the majority of burden of disorders such as depression [63]. Prevention interventions delivered early in the life course have the potential to avert the greatest burden.

It is important to acknowledge that the interventions included in the present review may not have been designed specifically for the university population; rather, the students may have simply been a convenient research sample. For studies clearly designed for university students in the tertiary setting (ie, exam anxiety [46-49], academic worry [58], stress [53], psychological distress [59], and adjustment and acculturation in international students [60]), there was no clear advantage for the development specifically within this population, with 3 [47,48,58] of the 8 studies finding at least 1 significant positive outcome compared with the control at postintervention.

However, the review also highlights that there are significant gaps in the current state of knowledge in this area. Very few studies have focused on the use of technology in the university setting for the treatment of mental disorders (n=3), and in very few cases did more than 1 study target the prevention or treatment of specific disorders. Most of the studies focused on anxiety symptoms or disorders. Moreover, some methodological problems were evident in the studies, with many studies failing to report sufficient information about randomization, or less frequently, suffering from inadequate randomization methods, few studies specifying a primary outcome, and most of the studies failing to undertake or report appropriate intent-to-treat analyses. It is also of note that none of the studies met criterion 6 of the EPOC quality scale. This criterion specifically refers to the blinding of outcome assessments (ie, “Score ‘low risk’ if the authors state explicitly that the primary outcome variables were assessed blindly, or the outcomes are objective, eg, length of hospital stay” [35]). This is an inherent problem with self-report population studies. Where outcome assessments are carried out using Internet-based self-report surveys and not clinical assessment, it is not possible to precisely meet this criterion because the participants (the outcome assessors) are themselves typically not blind to their assigned conditions in studies of psychological interventions in general as well as in Web-based research [64]. Study quality criteria designed specifically for Internet-based research would enable more accurate assessment of this characteristic. Nevertheless, it is imperative that studies explain in sufficient detail the methods they used to accurately assess study quality. In addition, more than half of the studies (14/27, 52%), including all those focused on conditions other than depression and anxiety, and almost all of the trials with universal samples (9/10, 90%) failed to provide sufficient data to calculate effect sizes. This information is vital to comparatively assess the effect of interventions accurately. All future studies in this area should endeavor to provide this information to enable appropriate comparison between studies. As would be expected, treatment trials recorded the largest effect sizes (alpha = 1.35) across all studies targeting depression and anxiety, or anxiety symptoms and disorders, followed by selective (alpha = 0.67) and indicated (alpha = 0.52) trials.

None of the studies reported information about cost-effectiveness. However, 1 study targeting depression and anxiety provided broad information about costs of dissemination for the workshop leader (US $2000/10-15 participants), coaching emails, and face-to-face booster sessions (US $55/hour) [41]. To the author’s knowledge, no trials of technology-based interventions in tertiary students have examined cost-effectiveness per se, although there has been 1 study of the cost-effectiveness of translating the English-language program MoodGYM and the website BluePages for delivery to Norwegian university students [65]. This is not unexpected despite the argument that Internet-based research is useful at reducing the cost of public health, a recent review of all health-related Internet-based interventions, including mental health, found a total of only 8 studies that reported on cost-effectiveness [66]. Further information about the cost-effectiveness of Internet-based research in university students is required.

Somewhat surprisingly, we failed to find an association between any of the characteristics of the studies or their methodological quality and whether they reported positive outcomes. In particular, it might have been predicted that there would be an association between length of intervention or number of intervention sessions and achieving a study outcome favoring the intervention group. The reason for this is unclear, but may reflect the heterogeneity of the studies across many variables and the small number of interventions for each condition, precluding investigation of the association for each condition separately.

### Limitations

There are some limitations to the present review that require consideration. Firstly, it is clear that some interventions were developed for university students (ie, the specific issues they face), and that others may have been simply tested in this population. Because of this, the included interventions may not have taken full advantage of the opportunities for technology-based interventions in a tertiary setting, which has important implications for the dissemination of these interventions within universities. However, some of the interventions were clearly university-specific with several of the anxiety interventions targeting student-focused problems, such as exam anxiety and stress in students, as well as adjustment and acculturation in international students. The present review searched 3 databases and it is possible that some relevant journals are not indexed by these databases. However, an attempt was made to address this by handsearching previous reviews, key papers, and the Beacon portal [67]. In addition, the restriction of the inclusion criteria to English-language journal papers may have introduced a level of bias into the present review.

### Conclusions

It is clear that further research is required in university settings to investigate the effectiveness of technological interventions for specific mental disorders in the tertiary student population, to compare the relative efficacy of and engagement with different types of technological intervention within a disorder and ultimately to evaluate the most appropriate means by which such interventions might routinely be implemented in university settings.

## Multimedia Appendix 1

PubMed search terms/search history.

## Multimedia Appendix 2

PRISMA checklist.

## Multimedia Appendix 3

Full data from the review.

## Abbreviations

APS: auto-photic stimulation
BAI: Beck Anxiety Inventory
BDI: Beck Depression Inventory
CBT: cognitive behavioral therapy
CD: compact disc
DVD: Digital Versatile Disc
EPOC: Effective Practice and Organisation of Care
ITT: intention-to-treat
MeSH: Medical Subject Headings
NHMRC: National Health and Medical Research Council
PRISMA: Preferred Reporting Items for Systematic Reviews and Meta-Analyses
SCT: social cognitive theory
STAI: State Trait Anxiety Inventory
TAFE: Technical and Further Education
