# Supplementary material for: Technology-Based Interventions for Mental Health in Tertiary Students: Systematic Review
Source: J Med Internet Res. 2013 May 27;15(5):e101. doi: 10.2196/jmir.2639 (PMC3668609; doi:10.2196/jmir.2639)
Supplement: Supplementary file 1 [file jmir_v15i5e101_app1.pdf]

PubMed

Search	Query	Items found
#38	Search **((#20) AND #35) AND #36** Limits: **Clinical Trial, Randomized Controlled Trial, Clinical Trial, Phase I, Clinical Trial, Phase II, Clinical Trial, Phase III, Clinical Trial, Phase IV, Controlled Clinical Trial**	729
#37	Search **((#20) AND #35) AND #36**	6112
#36	Search **computer OR computer-based OR cyber OR cyberspace OR electronic OR "electronic mail" OR email OR e-mail OR internet OR internet-based OR net OR online OR virtual OR web OR web-based OR webbased OR "world wide web" OR www OR phone OR telephone OR "smart phone" OR "cell phone" OR "cellular phone" OR iphone OR sms or "short message service" OR texting OR mobile OR "mobile phone" OR ipad OR tablet OR "smart device" OR digital OR "personal digital assistant" OR pda OR CD-ROM OR technology OR technologies OR technological OR "computers"[MeSH Terms] OR "electronics"[MeSH Terms] OR "electronic mail"[MeSH Terms] OR "internet"[MeSH Terms] OR "telephone"[MeSH Terms] OR "text messaging"[MeSH Terms] OR "cd-rom"[MeSH Terms] OR "technology"[MeSH Terms]** Field: **Title/Abstract**	1002012
#35	Search **"mental health" OR "mental disorder*" OR "mental disorders"[MeSH] OR psychiatr* OR "mental disease" OR "neuropsychiatric disorder*" OR psychopathology OR "domestic violence" OR fear OR "addiction" OR "alcohol dependence" OR "substance use" OR "substance abuse" OR intoxication OR "harmful use" OR withdrawal OR alcohol OR opioid* OR cannabinoids OR cannabis OR sedatives OR hypnotics OR cocaine OR stimulants OR caffeine OR hallucinogens OR tobacco OR "volatile solvents" OR "drug use" OR "drug abuse" OR "drug dependen*" OR "drug relapse" OR "drugs of dependence" OR "opioid dependen*" OR "smoking cessation" OR "smoking intervention" OR "tobacco control" OR "tobacco dependen*" OR "tobacco use" OR amphetamine OR "crystal meth*" OR GHB OR heroin OR ice OR marijuana OR MDMA OR methamphetamine* OR polysubstance OR phencyclidine OR Schizophrenia OR schizotypal OR schizophreniform OR schizoaffective OR schizoid OR paranoi* OR delusion* OR psychosis OR psychotic OR paraphrenia OR "affective disorder" OR "affective symptom*" OR "mood disorder*" OR depress* OR dysthymi* OR MDD OR CBT OR "cognitive behavior*" OR "cognitive behaviour*" OR IPT OR PST OR DBT OR psychotherapy OR stress OR "stress disorder" OR neurosis OR neurotic OR manic OR mania OR hypomania* OR cyclothymi* OR bipolar OR anxiety OR panic OR agoraphobi* OR "social phobia" OR "generalized anxiety disorder" OR GAD OR "obsessive compulsive" OR OCD OR "adjustment disorder" OR "separation anxiety" OR "post-traumatic stress" OR PTSD OR phobi* OR neurasthenia OR somatoform OR somatization OR "pain disorder*" OR hypochondria* OR hysteria OR dissociat* OR "depersonalization disorder" OR "personality disorder*" OR "borderline personality" OR BPD OR "antisocial personality" OR APD OR anankastik OR "dissocial personality" OR "dyssocial personality" OR "dependent personality" OR "anxious personality" OR "avoidant personality" OR histrionic OR narcissis* OR "passive-aggressive" OR "personality change" OR "eating disorder*" OR anorexia OR bulimia OR "body dysmorph*" OR**	2249339

“conversion disorder” OR “attention deficit” OR ADHD OR hyperkinetic OR hyperactive* OR impulsive* OR instability OR “Mood shifts” OR PDD-NOS OR autis* OR asperger* OR bully* OR “conduct disorder” OR “oppositional defiant” OR “emotional disorder” OR “separation disorder” OR tic OR tics OR tourette OR pica OR “stereotyped movement disorder*” OR “mild depressive disorder” OR internaliz* OR externaliz* OR internalis* OR externalis* OR “child abuse” OR “childhood disintegrative disorder” OR “pervasive developmental” OR compulsive OR delinquent OR aggress* OR “learning disorder*” OR “developmental disabilit*” OR “repetitive behaviours deficit” OR enuresis OR encopresis OR “mental retardation” OR “intellectual disability*” OR “behaviour* problem*” OR apprehension OR stuttering OR stammering OR cluttering OR “selective mutism” OR “attachment disorder” OR “sleep disorder*” OR insomnia OR hypersomnia OR dyssomnia OR parasomnia OR “sleep terror” OR sleepwalking OR gambl* OR pyromania OR kleptomania OR “sexual dysfunction” OR “tension headache” OR “sexual disorder” OR dyspareunia OR exhibitionism OR “orgasmic disorder” OR fetishism OR frotteurism OR “gender identity disorder” OR “impulse-control disorder” OR “intermittent explosive disorder” OR “erectile disorder” OR “nightmare” OR paraphilia OR pedophilia OR paedophilia OR “premature ejaculation” OR pyromania OR “sexual aversion disorder” OR “sexual masochism” OR “sexual sadism” OR sadomasochism OR “sexual maturation disorder” OR “transvestic fetishism” OR “egodystonic sexual orientation” OR “sexual relationship disorder” OR “sexual arousal disorder” OR dyspareunia OR trichotillomania OR vaginismus OR voyeurism OR suicid* OR self injur* OR “self harm” OR violen* OR offend* OR “Factitious disorder” OR munchausen OR “ganser syndrome” Field: **Title/Abstract**

#34	Search **“mental health” OR “mental disorder*” OR “mental disorders”[MeSH] OR psychiatr* OR “mental disease” OR “neuropsychiatric disorder*” OR psychopathology OR “domestic violence” OR fear OR “addiction” OR “alcohol dependence” OR “substance use” OR “substance abuse” OR intoxication OR “harmful use” OR withdrawal OR alcohol OR opioid* OR cannabinoids OR cannabis OR sedatives OR hypnotics OR cocaine OR stimulants OR caffeine OR hallucinogens OR tobacco OR “volatile solvents” OR “drug use” OR “drug abuse” OR “drug dependen*” OR “drug relapse” OR “drugs of dependence” OR “opioid dependen*” OR “smoking cessation” OR “smoking intervention” OR “tobacco control” OR “tobacco dependen*” OR “tobacco use” OR amphetamine OR “crystal meth*” OR GHB OR heroin OR ice OR marijuana OR MDMA OR methamphetamine* OR polysubstance OR phencyclidine OR Schizophrenia OR schizotypal OR schizophreniform OR schizoaffective OR schizoid OR paranoi* OR delusion* OR psychosis OR psychotic OR paraphrenia OR “affective disorder” OR “affective symptom*” OR “mood disorder*” OR depress* OR dysthymi* OR MDD OR CBT OR “cognitive behavior*” OR “cognitive behaviour*” OR IPT OR PST OR DBT OR psychotherapy OR stress OR “stress disorder” OR neurosis OR neurotic OR manic OR mania OR hypomania* OR cyclothymi* OR bipolar OR anxiety OR panic OR agoraphobi* OR “social phobia” OR “generalized anxiety disorder” OR GAD OR “obsessive compulsive” OR OCD OR “adjustment disorder” OR “separation anxiety” OR “post-traumatic stress” OR PTSD OR phobi* OR neurasthenia OR somatoform OR somatization OR “pain disorder*” OR hypochondria* OR hysteria OR dissociat* OR “depersonalization disorder” OR “personality disorder*” OR “borderline personality” OR BPD OR “antisocial**	118669

	**personality" OR APD OR anankastik OR "dissocial personality" OR "dyssocial personality" OR "dependent personality" OR "anxious personality" OR "avoidant personality" OR histrionic OR narcissis* OR "passive-aggressive" OR "personality change" OR "eating disorder*" OR anorexia OR bulimia OR "body dysmorph*" OR "conversion disorder" OR "attention deficit" OR ADHD OR hyperkinetic OR hyperactive* OR impulsive* OR instability OR "Mood shifts" OR PDD-NOS OR autis* OR asperger* OR bully* OR "conduct disorder" OR "oppositional defiant" OR "emotional disorder" OR "separation disorder" OR tic OR tics OR tourette OR pica OR "stereotyped movement disorder*" OR "mild depressive disorder" OR internaliz* OR externaliz* OR internalis* OR externalis* OR "child abuse" OR "childhood disintegrative disorder" OR "pervasive developmental" OR compulsive OR delinquent OR aggress* OR "learning disorder*" OR "developmental disabilit*" OR "repetitive behaviours deficit" OR enuresis OR encopresis OR "mental retardation" OR "intellectual disability*" OR "behaviour* problem*" OR apprehension OR stuttering OR stammering OR cluttering OR "selective mutism" OR "attachment disorder" OR "sleep disorder*" OR insomnia OR hypersomnia OR dyssomnia OR parasomnia OR "sleep terror" OR sleepwalking OR gambl* OR pyromania OR kleptomania OR "sexual dysfunction" OR "tension headache" OR "sexual disorder" OR dyspareunia OR exhibitionism OR "orgasmic disorder" OR fetishism OR frotteurism OR "gender identity disorder" OR "impulse-control disorder" OR "intermittent explosive disorder" OR "erectile disorder" OR "nightmare" OR paraphilia OR pedophilia OR paedophilia OR "premature ejaculation" OR pyromania OR "sexual aversion disorder" OR "sexual masochism" OR "sexual sadism" OR sadomasochism OR "sexual maturation disorder" OR "transvestic fetishism" OR "egodystonic sexual orientation" OR "sexual relationship disorder" OR "sexual arousal disorder" OR dyspareunia OR trichotillomania OR vaginismus OR voyeurism OR suicid* OR self injur* OR "self harm" OR violen* OR offend* OR "Factitious disorder" OR munchausen OR "ganser syndrome"** Limits: **Clinical Trial, Randomized Controlled Trial, Clinical Trial, Phase I, Clinical Trial, Phase II, Clinical Trial, Phase III, Clinical Trial, Phase IV, Controlled Clinical Trial** Field: **Title/Abstract**	
#33	Search **((#20) AND #22) AND #31** Limits: **Clinical Trial, Randomized Controlled Trial, Clinical Trial, Phase I, Clinical Trial, Phase II, Clinical Trial, Phase III, Clinical Trial, Phase IV, Controlled Clinical Trial**	735
#31	Search **computer OR computer-based OR cyber OR cyberspace OR electronic OR "electronic mail" OR email OR e-mail OR internet OR internet-based OR net OR online OR virtual OR web OR web-based OR webbased OR "world wide web" OR www OR phone OR telephone OR "smart phone" OR "cell phone" OR iphone OR sms or "short message service" OR texting OR mobile OR "mobile phone" OR ipad OR tablet OR "smart device" OR digital OR "personal digital assistant" OR pda OR CD-ROM OR technology OR technologies OR technological OR "computers"[MeSH Terms] OR "electronics"[MeSH Terms] OR "electronic mail"[MeSH Terms] OR "internet"[MeSH Terms] OR "telephone"[MeSH Terms] OR "text messaging"[MeSH Terms] OR "cd-rom"[MeSH Terms] OR "technology"[MeSH Terms]** Field: **Title/Abstract**	1002012
#32	Search **((#20) AND #22) AND #31**	6189
#30	Search **technology**	650057

#29	Search **technology** Limits: **Clinical Trial, Randomized Controlled Trial, Clinical Trial, Phase I, Clinical Trial, Phase II, Clinical Trial, Phase III, Clinical Trial, Phase IV, Controlled Clinical Trial**	13408
#28	Search **#25** Limits: **Clinical Trial, Randomized Controlled Trial, Clinical Trial, Phase I, Clinical Trial, Phase II, Clinical Trial, Phase III, Clinical Trial, Phase IV, Controlled Clinical Trial**	600
#27	Search **#25**	4795
#26	Search **((#20) AND #22) AND #23** Limits: **Randomized Controlled Trial**	423
#25	Search **((#20) AND #22) AND #23**	4795
#24	Search **computer OR cyber OR electronic OR “electronic mail” OR email OR e-mail OR internet OR net OR online OR virtual OR web OR “world wide web” OR www OR phone OR sms or “short message service” OR texting OR “text message” OR “text messaging” OR mobile OR ipad OR tablet OR “smart device” OR digital OR “personal digital assistant” OR pda OR CD-ROM OR "computers"[MeSH Terms] OR "electronics"[MeSH Terms] OR "electronic mail"[MeSH Terms] OR "internet"[MeSH Terms] OR "telephone"[MeSH Terms] OR "text messaging"[MeSH Terms] OR "cd-rom"[MeSH Terms]** Field: **Title/Abstract**	601455
#23	Search **computer OR computer-based OR cyber OR cyberspace OR electronic OR “electronic mail” OR email OR e-mail OR internet OR internet-based OR net OR online OR virtual OR web OR web-based OR webbased OR “world wide web” OR www OR phone OR telephone OR “smart phone” OR “cell phone” OR iphone OR sms or “short message service” OR texting OR “text message” OR “text messaging” OR mobile OR “mobile phone” OR ipad OR tablet OR “smart device” OR digital OR “personal digital assistant” OR pda OR CD-ROM OR "computers"[MeSH Terms] OR "electronics"[MeSH Terms] OR "electronic mail"[MeSH Terms] OR "internet"[MeSH Terms] OR "telephone"[MeSH Terms] OR "text messaging"[MeSH Terms] OR "cd-rom"[MeSH Terms]** Field: **Title/Abstract**	626861
#22	Search **“mental health” OR “mental disorder*” OR “mental disorders”[MeSH] OR psychiatr* OR “mental disease” OR “neuropsychiatric disorder*” OR psychopathology OR “domestic violence” OR fear OR “addiction” OR “alcohol dependence” OR “substance use” OR “substance abuse” OR intoxication OR harmful use OR withdrawal OR alcohol OR opioid* OR cannabinoids OR cannabis OR sedatives OR hypnotics OR cocaine OR stimulants OR caffeine OR hallucinogens OR tobacco OR “volatile solvents” OR “drug use” OR “drug abuse” OR “drug dependen*” OR “drug relapse” OR “drugs of dependence” OR “opioid dependen*” OR “smoking cessation” OR “smoking intervention” OR “tobacco control” OR “tobacco dependen*” OR “tobacco use” OR amphetamine OR “crystal meth*” OR GHB OR heroin OR ice OR marijuana OR MDMA OR methamphetamine* OR polysubstance OR phencyclidine OR Schizophrenia OR schizotypal OR schizophreniform OR schizoaffective OR schizoid OR paranoi* OR delusion* OR psychosis OR psychotic OR paraphrenia OR “affective disorder” OR “affective symptom*” OR “mood disorder*” OR depress* OR dysthymi* OR MDD OR CBT OR “cognitive behavior*” OR “cognitive behaviour*” OR IPT OR PST OR DBT OR**	2269376

	**psychotherapy OR stress OR “stress disorder” OR neurosis OR neurotic OR manic OR mania OR hypomania* OR cyclothymi* OR bipolar OR anxiety OR panic OR agoraphobi* OR “social phobia” OR “generalized anxiety disorder” OR GAD OR “obsessive compulsive” OR OCD OR “adjustment disorder” OR “separation anxiety” OR “post-traumatic stress” OR PTSD OR phobi* OR neurasthenia OR somatoform OR somatization OR “pain disorder*” OR hypochondria* OR hysteria OR dissociat* OR “depersonalization disorder” OR “personality disorder*” OR “borderline personality” OR BPD OR “antisocial personality” OR APD OR anankastik OR “dissocial personality” OR “dyssocial personality” OR “dependent personality” OR “anxious personality” OR “avoidant personality” OR histrionic OR narcissis* OR “passive-aggressive” OR “personality change” OR “eating disorder*” OR anorexia OR bulimia OR “body dysmorph*” OR “conversion disorder” OR “attention deficit” OR ADHD OR hyperkinetic OR hyperactive* OR impulsive* OR instability OR “Mood shifts” OR PDD-NOS OR autis* OR asperger* OR bully* OR “conduct disorder” OR “oppositional defiant” OR “emotional disorder” OR “separation disorder” OR tic OR tics OR tourette OR pica OR “stereotyped movement disorder*” OR “mild depressive disorder” OR internaliz* OR externaliz* OR internalis* OR externalis* OR “child abuse” OR “childhood disintegrative disorder” OR “pervasive developmental” OR compulsive OR delinquent OR aggress* OR “learning disorder*” OR “developmental disabilit*” OR “repetitive behaviours deficit” OR enuresis OR encopresis OR “mental retardation” OR “intellectual disability*” OR “behaviour* problem*” OR apprehension OR stuttering OR stammering OR cluttering OR “selective mutism” OR “attachment disorder” OR “sleep disorder*” OR insomnia OR hypersomnia OR dyssomnia OR parasomnia OR “sleep terror” OR sleepwalking OR gambl* OR pyromania OR kleptomania OR “sexual dysfunction” OR “tension headache” OR “sexual disorder” OR dyspareunia OR exhibitionism OR “orgasmic disorder” OR fetishism OR frotteurism OR “gender identity disorder” OR “impulse-control disorder” OR “intermittent explosive disorder” OR “erectile disorder” OR “nightmare” OR paraphilia OR pedophilia OR paedophilia OR “premature ejaculation” OR pyromania OR “sexual aversion disorder” OR “sexual masochism” OR “sexual sadism” OR sadomasochism OR “sexual maturation disorder” OR “transvestic fetishism” OR “egodystonic sexual orientation” OR “sexual relationship disorder” OR “sexual arousal disorder” OR dyspareunia OR trichotillomania OR vaginismus OR voyeurism OR suicid* OR self injur* OR “self harm” OR violen* OR offend* OR “Factitious disorder” OR munchausen OR “ganser syndrome”** Field: **Title/Abstract**	
#20	Search **university[Title/Abstract] OR universities[Title/Abstract] OR university-based[Title/Abstract] OR tertiary[Title/Abstract] OR college[Title/Abstract] OR colleges[Title/Abstract] OR student[Title/Abstract] OR students[Title/Abstract] OR undergraduate[Title/Abstract] OR undergraduates[Title/Abstract] OR dorm[Title/Abstract] OR dorms[Title/Abstract] OR fraternity[Title/Abstract] OR fraternities[Title/Abstract] OR sorority[Title/Abstract] OR sororities[Title/Abstract] OR academic[Title/Abstract] OR academics[Title/Abstract] OR academia[Title/Abstract] OR scholar[Title/Abstract] OR scholars[Title/Abstract] OR "students"[MeSH Terms] OR "universities"[MeSH Terms]** Field: **Title/Abstract**	494182
#19	Search **university[Title/Abstract] OR universities[Title/Abstract] OR tertiary[Title/Abstract] OR college[Title/Abstract] OR student[Title/Abstract] OR**	445749

undergraduate[Title/Abstract] OR dorm[Title/Abstract] OR fraternity[Title/Abstract] OR fraternities[Title/Abstract] OR sorority[Title/Abstract] OR sororities[Title/Abstract] OR academic[Title/Abstract] OR academia[Title/Abstract] OR scholar[Title/Abstract] OR "students"[MeSH Terms] OR "universities"[MeSH Terms] Field: **Title/Abstract**
