# Supplementary material for: Technology-Based Interventions for Mental Health in Tertiary Students: Systematic Review
Source: J Med Internet Res. 2013 May 27;15(5):e101. doi: 10.2196/jmir.2639 (PMC3668609; doi:10.2196/jmir.2639)
Supplement: Supplementary file 3 [file jmir_v15i5e101_app3.pdf]

Study<br>Country<br>Disorder or symptoms targeted	Participants<br>(# randomized)<br>(# analyzed, if not ITT)	Age<br>(M, SD)<br>Sex (%F)	Intervention description	Type	Technology used	Distal?	Human contac t?	ITT?	Quality rating	Primary outcome/ Measurement points	Significant interaction between time and group, favoring intervention group?	Effect size between intervention and the control (Hedge's *g* unless otherwise specified)
**Depression & Anxiety (n = 7)**												
Braithwaite & Fincham (2007) [36]<br><br>USA<br><br>Depression & Anxiety symptoms	N = 91, I^1 = 33, I^2 = 31, C = 27 (Randomized)<br>Analyzed sample size not reported<br>Introductory psychology students in a romantic relationship for 4+ months<br>Recruitment: recruited from undergraduate psychology subject pool	M = 19.34<br>SD = not reported<br>F = 59%	I^1 = 1x 1 hour session of e-PREP: relationship focused skills training combating dynamic risk factors for relationship stress (communication, problem-solving) + 7 weekly e-mails<br>I^2 = 1 x 1 hour session of CBASP: techniques for analyzing and changing maladaptive thinking patterns/behavior (CBT) + 7 weekly e-mails<br>C = 1 x 1 hour session of descriptive information about anxiety, depression and relationships + 7 weekly e-mails	Universal	I^1 = Computer program + Internet<br>I^2 = Computer program + Internet<br>C = Computer program + Internet	I^1 = Combined<br>I^2 = Combined<br>C = Combined	I^1 = SA<br>I^2 = SA<br>C = SA	Not specified	5/9	1. BAI<br>2. BDI<br><br>Baseline<br>8 weeks	8 weeks:<br>1. I^1: No<br>I^2: Yes<br>2. I^1: Yes<br>I^2: Yes	Insufficient data to calculate effect sizes
Braithwaite & Fincham (2009) [37]<br><br>USA<br><br>Depression & Anxiety symptoms	N = 77, I = 38, C = 39<br>Introductory psychology students in a romantic relationship for 4+ months<br>Recruitment: announcements	M = 19.4<br>SD = not reported<br>F = 71%	I = 1 x 1 hour session of e-PREP: relationship focused skills training combating dynamic risk factors for relationship stress (communication, problem-solving) + 7 weekly e-mails<br>C = 1 x 1 hour session of descriptive information about anxiety, depression and relationships + 7 weekly e-mails	Universal	I = Computer program + Internet<br>C = Computer program + Internet	I = Combined<br>C = Combined	I = SA<br>C = SA	Yes<br><br>MLE	6/9	1. BAI<br>2. BDI<br><br>Baseline<br>8 weeks<br>10 months	8 weeks:<br>1. No<br>2. No<br>10 months:<br>1. Yes<br>2. No	8 weeks:<br>1. -1.52<br>2. 0.05<br>10 months:<br>1. 1.01<br>2. 1.29
Cukrowicz & Joiner (2007) [38]	N = 238 (Randomized)<br>N = 152, I = 81, C	M = 19.2<br>SD = 1.9<br>F = 73.7%	I = 2 hour educational information + CBT techniques + take	Indicated<br><br>(Mild	I = Computer program +	I = Combined<br>C = Combined	I = SA<br>C = SA	No	5/9	1. BAI<br>2. BDI	8 weeks:<br>1. Yes<br>2. Yes	8 weeks:<br>1. 0.58<br>2. 0.50

USA<br><br>Depression & Anxiety symptoms	= 71 (Analyzed)<br>Undergraduate psychology students with mild symptoms of depression and anxiety<br>Recruitment: Students in psychology department "participant pool" participated for course credit.	(final sample)	home slides and copies of SA worksheets.<br>C = 2 hour educational information on anxiety and depression + take home copy of information slides.<br><br>Both groups received email reminders of content learned once a week for 8 weeks.	symptoms of depression and anxiety: BAI ≤ 18 + BDI ≤ 19)	Internet<br>C = Computer program + Internet					Baseline<br>8 weeks		
Ellis et al. (2011) [39]<br><br>AUS<br><br>Depression & Anxiety symptoms	N = 39, I^1 = 13, I^2 = 13, C = 13<br>Undergraduate students with low-moderate psychological distress<br>Recruitment: students from Department of Psychology and Faculty of Health Sciences	M = 19.67<br>SD = 1.66<br>F = 77%	I^1 = "MoodGYM": 3 x weekly 1 hour sessions of online CBT, 5 modules + therapist guidance<br>I^2 = "Mood Garden": 3 x weekly 1 hour sessions of online peer-based support and information + therapist guidance<br>C = No intervention	Selective<br><br>Low to moderate levels of psychological distress on K-10	I^1 = Internet<br>I^2 = Internet<br>C = N/A	I^1 = No<br>I^2 = No<br>C = N/A	I^1 = MC<br>I^2 = MC<br>C = N/A	Yes<br><br>No dropout	5/9	1. DASS (anxiety)<br>2. DASS (depression)<br><br>Baseline<br>5 weeks	5 weeks:<br>1. I^1 : Yes<br>I^2 : Yes<br>2. I^1 : No<br>I^2 : No	5 weeks:<br>1. I^1 : 1.0<br>I^2 : 0.96<br>2. I^1 : 0.46<br>I^2 : 0.63
Mailey et al. (2010) [40]<br><br>USA<br><br>Depression & Anxiety symptoms	N = 51, I = 26, C = 25 (Randomized)<br>N = 37, I = 23, C = 24 (Analyzed)<br>University students currently receiving counseling<br>Recruitment: Flyers distributed to counseling centers and advertisements in the daily university newspaper	M = 25.0<br>SD = NR<br>F = 68.1%	I = 10-week internet-based physical activity intervention including pedometer, email tracking, website comprising 4 modules based on social cognitive theory + 2 meetings with their physical activity counselors. C = Waitlist control and treatment as usual (mental health counseling).	Treatment<br><br>Students must have been registered for and receiving counseling at the time of recruitment.	I = Internet + computer software<br>C = None	I = Combined<br>C = No	I = TA + MC<br>C = TA	No	3/9	1. STAI<br>2. BDI<br><br>Baseline<br>10 weeks	10 weeks:<br>1. No<br>2. No	10 weeks:<br>1. 0.22<br>2. 0.13
Seligman et al. (2007) [41]	N = 240, I = 113, C = 127 (Randomized)	M = 18<br>SD = not reported	I = 2 hours x 8 weeks of classroom-based group workshop teaching CBT	Selective<br><br>Participant	I = Internet<br>C = N/A	I = Combined<br>C = N/A	I = TA<br>C = N/A	No	4/9	1. BDI<br>2. BAI	6 weeks:<br>1. Yes<br>2. Yes	6 weeks:<br>1. 0.66<br>2. 0.34

USA<br><br>Depression<br>&<br>Anxiety symptoms	N = 212, I = 92, C = 120<br>(Analyzed at follow-up)<br>First year undergraduate students at risk for depression<br>Recruitment: BDI mailed to all incoming first year students	F = 65%	strategies + web-based homework + e-mail coaching for 6 months + face-to-face booster session<br>C = No intervention	s at risk for depression (score of 9-24 on BDI)						Baseline<br>8 weeks<br>6 months<br>8 months	6 months:<br>1. Yes<br>2. Yes<br>8 months:<br>1. No<br>2. No	6 months:<br>1. 0.63<br>2. 0.47<br>8 months:<br>1. 0.23<br>2. 0.13
Sethi et al. (2010) [42]<br><br>AUS<br><br>Depression<br>&<br>Anxiety symptoms	N = 38, I^1 = 10, I^2 = 9, I^3 = 9, C = 10<br>First year undergraduate students with low to moderate levels of depression or anxiety<br>Recruitment: Short verbal advertisements in lectures	M = 19.47<br>SD = 1.57<br>F = 65.8%	I^1 = 3 x weekly sessions of face-to-face CBT<br>I^2 = 3 x weekly sessions of "MoodGYM": automated online CBT<br>I^3 = 3 x weekly sessions of half face-to-face CBT and half automated online CBT<br>C = No intervention	Selective<br><br>Low to moderate levels of depression or anxiety on the DASS	I^1 = None<br>I^2 = Internet<br>I^3 = Internet<br>C = N/A	I^1 = No<br>I^2 = No<br>I^3 = No<br>C = N/A	I^1 = TA<br>I^2 = MC<br>I^3 = TA + MC<br>C = N/A	Yes<br><br>No dropout	3/9	1. DASS (depression)<br>2. DASS (anxiety)<br>3. K-10<br><br>Baseline<br>4 weeks	4 weeks:<br>1. I^1 : NR<br>I^2 : No<br>I^3 : Yes<br>2. I^1 : NR<br>I^2 : Yes<br>I^3 : Yes<br>3. I^1 : NR<br>I^2 : Yes<br>I^3 : Yes	4 weeks:<br>1. I^1 : 1.99<br>I^2 : -0.07<br>I^3 : 3.04<br>2. I^1 : 1.62<br>I^2 : 1.30<br>I^3 : 3.37<br>3. I^1 : 6.18<br>I^2 : 2.78<br>I^3 : 7.83

Notes: SA = self-administered therapy, PSH = predominantly self-help, MC = minimal-contact therapy, TA = predominantly therapist administered treatments, MLE = Maximum Likelihood Estimation, ED = Eating disorder, BMI = Body Mass Index, PB = Post baseline, PI = Post-intervention, ID = Insufficient data, NR = Not reported, LOCF = Last observation carried forward, GLMM = General linear mixed models, MLE = Maximum likelihood estimation.

Measures: BAI = Beck Anxiety Inventory, BDI = Beck Depression Inventory, DASS = Depression, Anxiety and Stress Scales, STAI = State Trait Anxiety Inventory, K-10 = Kessler Psychological Distress Scale, Fear (TB) = Target behavior (fear of social situations), Avoid (TB) = Target behavior (avoidance of social situations), Belief (TB) = Target behavior (belief in catastrophic thoughts regarding social situations), TAI = Test Anxiety Inventory, PSS = Perceived Stress Scale, ASI = Anxiety Sensitivity Index, IES = Impact of Events Scale, AD-ACL = Momentary Moods States Checklist, AQ = Acrophobia Questionnaire, AWQ = Academic Worry Questionnaire, PSWQ = Penn State Worry Questionnaire, CORE-OM = Clinical Outcomes in Routine Evaluation – Outcome Measure.

Study<br>Country<br>Disorder or symptoms targeted	Participants<br>(# randomized)<br>(# analyzed, if not ITT)	Age<br>(M, SD)<br>Sex (%F)	Intervention description	Type	Technology used	Distal?	Human contact?	ITT?	Quality rating	Primary outcome/ Measurement points	Significant interaction between time and group, favoring intervention group?	Effect size between intervention and the control (Hedge's *g* unless otherwise specified)
**Anxiety symptoms (n = 4)**												
Grassi et al. (2009) [43]<br><br>ITA<br><br>Anxiety symptoms	N = 120, I^1 = 30, I^2 = 30, I^3 = 30 , C = 30<br>Analyzed sample size not reported<br>University students who commute by train to university.<br>Recruitment: Conducted on the Varese-Milano train route (70 minute trip).	M = 23.27<br>SD = 1.38<br>F = 50%	I^1 = Video of 4 parts of a virtual island (2 day, 2 night scenes) + audio narrative guiding island exploration + relaxation exercises.<br>I^2 = Video of 4 parts of virtual island alone.<br>I^3 = Audio narrative guiding island exploration alone.<br><br>All interventions were 4 x 10 minute sessions over 2 days.<br><br>C = No intervention	Universal	I^1 = Mobile phone<br>I^2 = Mobile phone<br>I^3 = MP3 player<br>C = N/A	I^1 = Yes<br>I^2 = Yes<br>I^3 = Yes<br>C = N/A	I^1 = SA<br>I^2 = SA<br>I^3 = SA<br>C = N/A	NR	3/9	1. STAI (state)<br><br>Day 1 AM<br>Day 1 PM<br>Day 2 AM<br>Day 2 PM	Significant within-group decline in STAI-State for I^1 over time.<br><br>I^2, I^3, and C did not show significant within-group decline.<br><br>Insufficient information to determine effectiveness of each intervention relative to the control at each measurement point	Insufficient information to determine effectiveness of each intervention relative to the control at each measurement point
Kenardy et al. (2003) [31]<br>Kenardy et al. (2006) [32]<br><br>AUS<br><br>Anxiety symptoms	N = 83, I = 43, C = 40<br>(Randomized)<br>N = 74, I = 36, C = 38<br>(Analyzed)<br>First year psychology students scoring in the top 3rd on the ASI<br>Recruitment: 1sy year psychology students screened and received course credit for participation	M = 20.73<br>SD = 6.29<br>F = 61.7%	I = 6 weekly sessions of the "online anxiety prevention program" including psychoeducation, relaxation training, interoceptive exposure and cognitive restructuring<br>C = Wait-list control	Indicated<br><br>High anxiety sensitivity (top 3rd of scores on ASI)	I = Internet<br>C = N/A	I = Yes<br>C = N/A	I = SA<br>C = N/A	No	5/9	1. ASI<br><br>Baseline<br>6 weeks<br>6 months	6 weeks:<br>1. No<br>6 months:<br>1. No	6 weeks:<br>1. 0.30<br>6 months<br>0.35
Stephens (1992) [44]	N = 159<br>(Randomized)	43% 18-24<br>SD = not	I^1 = 14 x 15 mins sessions over 4 weeks	Universal	I^1 = Audio<br>I^2 = Audio	I^1 = Yes<br>I^2 = Yes	I^1 = SA<br>I^2 = SA	No	4/9	1. STAI (state)	4 weeks:<br>1. I^1 : Yes	Insufficient data to

USA<br><br>Anxiety symptoms	N = 100, I^1 = 31, I^2 = 31, C = 38 (Analyzed)<br>First year female nursing students<br>Recruitment: Not reported	reported<br>F = 100%	of exposure therapy by imagery audiotape<br>I^2 = 14 x 20 mins sessions over 4 weeks of exposure therapy by imagery audiotape + progressive muscular relaxation<br>C = No intervention		C = N/A	C = N/A	C = N/A			Baseline<br>4 weeks	I^2 : Yes	calculate effect sizes
Villani & Riva (2008) [45]<br><br>ITA<br><br>Anxiety symptoms	N = 60, I^1 = 15, I^2 = 15, I^3 = 15, C = 15<br>Analyzed sample size not reported<br>University students<br>Recruitment: Volunteered	M = 24.52<br>SD = 1.75<br>F = 50%	I^1 = 2 sessions of virtual reality relaxation experiences and environment, supported by a relaxing narrative<br>I^2 = 2 sessions of a relaxation scene via video, supported by a relaxing narrative<br>I^3 = 2 sessions of relaxation via audiotape with a relaxing narrative<br><br>All interventions were completed within 1 week.1<br><br>C = No intervention	Universal	I^1 = Virtual reality<br>I^2 = Video<br>I^3 = Audio<br>C = N/A	I^1 = No<br>I^2 = No<br>I^3 = No<br>C = N/A	I^1 = SA<br>I^2 = SA<br>I^3 = SA<br>C = N/A	NR	4/9	1. STAI<br><br>Baseline<br>After session 1<br>Before session 2<br>After session 2	After session 1<br>1. I^1 : No<br>I^2 : No<br>I^3 : No<br>Before session 2<br>1. I^1 : No<br>I^2 : No<br>I^3 : No<br>After session 2<br>1. I^1 : No<br>I^2 : No<br>I^3 : No	Insufficient data to calculate effect sizes
**Examination anxiety (n = 4)**												
Buglione et al. (1990) [46]<br><br>USA<br><br>Examination anxiety	N = 50 (Randomized)<br>N = 36, I^1 = 16, I^2 = 20 (Analyzed)<br>Students scoring above the 60th percentile on the TAI<br>Recruitment: All students prior to starting first year were screened. Letters sent to eligible participants, as well as flyers and advertisements	M = 19<br>SD= not reported<br>F = 42% (completers)	I^1 = Maximum of 10 x 1 hour sessions over 6 wks of "Coping with tests": computer-assisted systematic desensitization + audiotaped Jacobsonian relaxation<br>I^2 = 6 weeks of group-based systematic desensitization + rational emotive discussion + audiotaped Jacobsonian relaxation	Indicated<br><br>A score above the 60th percentile on the TAI	I^1 = Computer program + audio<br>I^2 = Audio	I^1 = No<br>I^2 = No	I^1 = SA<br>I^2 = TA	No	Not rated (no control group)	1. TAI<br><br>Baseline<br>~6 weeks	6 weeks:<br>NO CONTROL<br>1. I^1 = I^2<br><br>Both groups showed within-group change	Insufficient data to calculate effect sizes
Grassi et al.	N = 75, I^1 = NR, I^2	M = 20.86	I^1 = Stress Inoculation	Universal	I^1 = Mobile	I^1 = Yes	I^1 = SA	NR	4/9	1. STAI (state)	Pre-Post 1	Insufficient

(2011) [47]<br><br>ITA<br><br>Examination anxiety	= NR, I^3 = NR, I^4 = NR, C = NR<br>(Randomized)<br>Analyzed sample size not reported<br>Female university students.<br>Recruitment: NR	SD = 1.27<br>F = 100%	Training (SIT) via audio and video narrative to manage exam anxiety and enhance coping strategies + muscular relaxation competencies<br>I^2 = SIT content via DVD.<br>I^3 = SIT content audio only via MP3.<br>I^4 = SIT content audio only via CD.<br><br>All interventions were 6 sessions (time NR) delivered over 6 days.<br><br>C = No intervention		phone<br>I^2 = DVD<br>I^3 = MP3<br>I^4 = CD<br>C = N/A	I^2 = Yes<br>I^3 = Yes<br>I^4 = Yes<br>C = N/A	I^2 = SA<br>I^3 = SA<br>I^4 = SA<br>C = N/A			Pre and post each of the 6 sessions (12 points)<br><br>Pre-Post 1<br>Pre-Post 2<br>Pre-Post 3<br>Pre-Post 4<br>Pre-Post 5<br>Pre-Post 6	I^1 : Yes, I^2 : Yes , I^3 : Yes, I^4 : Yes<br><br>Pre-Post 2<br>I^1 : Yes, I^2 : Yes , I^3 : Yes, I^4 : Yes<br><br>Pre-Post 3<br>I^1 : Yes, I^2 :Yes , I^3 : Yes, I^4 : Yes<br><br>Pre-Post 4<br>I^1 : Yes, I^2 : Yes , I^3 : Yes, I^4 : Yes<br><br>Pre-Post 5<br>I^1 : Yes, I^2 : Yes , I^3 : Yes, I^4 : Yes<br><br>Pre-Post 6<br>I^1 : Yes, I^2 : Yes , I^3 : Yes, I^4 : Yes<br><br>Insufficient data to calculate effect sizes	data to calculate effect sizes
Orbach et al. (2007) [48]<br><br>GBR<br><br>Examination anxiety	N = 90, I = 47, C = 43<br>(Randomized)<br>N = 58, I = 30, C = 28<br>(Analyzed)<br>University students who experience anxiety when preparing for or taking exams<br>Recruitment: Invited students who experience exam anxiety via university-wide e-mail	M(I) = 24.72<br>SD(I) = 6.89<br>M(C) = 22.54<br>SD(C) = 5.71<br>F(I) = 60%<br>F(C) = 86%	I = 6 x 30 min modules of online CBT, progressive muscle relaxation, rational thinking, and a thought diary<br>C = 4 x 30 mins of education about text anxiety, relaxation, puzzles and a thought diary<br><br>Prior to receiving the intervention, both conditions received a demonstration of the program by the experimenter. Interventions were delivered over 6 weeks.	Selective<br><br>Self-reported anxiety when preparing for or taking exams	I = Internet<br>C = Internet	I = Yes<br>C = Yes	I = PSH<br>C = N/A	No	6/9	1. TAI<br><br>Baseline<br>6-8 weeks<br>4 months post-intervention	6-8 weeks:<br>1. Yes<br>4 months post-intervention:<br>No TAI data at follow-up	6-8 weeks:<br>1. 0.67<br>4 months post-intervention:<br>No TAI data at follow-up
Riva e al.	N = 30	M = 23.48	I^1 = 6 sessions of stress	Universal	I^1 = Audio	I^1 = Yes	I^1 = SA	Not	1/9	1. STAI	Insufficient data	Insufficient

(2007) [49]<br><br>ITA<br><br>Examination anxiety	Female students due to perform an exam within a week<br>Recruitment: Not reported	SD = 1.24<br>F = 100%	inoculation training via audio CD. Targets psychophysiological reactions to exams and coping strategies<br>I^2 = 6 sessions of stress inoculation training via audio delivered through portable MP3<br>I^3 = 6 sessions of stress inoculation training via audio and video DVD<br>I^4 = 6 sessions of stress inoculation training via audio and video via UMTS 3G mobile phone<br><br>All interventions conducted within 1 week period.<br><br>C = No intervention		CD<br>I^2 = Audio MP3<br>I^3 = Audio/video DVD<br>I^4 = Audio/video mobile phone<br>C = N/A	I^2 = Yes<br>I^3 = Yes<br>I^4 = Yes<br>C = N/A	I^2 = SA<br>I^3 = SA<br>I^4 = SA<br>C = N/A	reported /unclear		 Baseline<br>Before exam<br>After exam	to determine effectiveness of each intervention relative to the control	data to calculate effect sizes
**Specific phobia (n = 3)**												
Hoffman et al. (2003) [50]<br><br>USA<br><br>Spider phobia	N = 36, I^1 = 12, I^2 = 12, C = 12<br>Students in Introductory Psychology class with fear of spiders<br>Recruitment: Screening survey conducted in classes and eligible students invited to participate	M = 18.66<br>SD = NR<br>F = 80.6%	I^1 = 3 x 1 hour one-on – one clinical virtual reality exposure therapy sessions with a clinical psychologist including with their cyberhand 1) attempting to approach within arm's reach of virtual spider, 2) picking up the bucket containing spider, 3) 'touching' spider until habituated<br>I^2 = 3 x 1 hour one-on-one clinical virtual reality exposure therapy sessions with clinical psychologist + tactile stimulation of their real hand of toy spider during session 3 (above)<br>C = 15 minutes in virtual reality scenario without spiders	Indicated<br><br>(Fear of spiders questionnaire > 1 SD above class M)	I^1 = Virtual reality<br>I^2 = Virtual reality<br><br>All virtual reality was conducted with Silicon Graphics Octane MXE with Octane Channel Option coupled with wide field of view (40° vertical x 105° horizontal with 40° overlap) head mounted	I^1 = No<br>I^2 = No<br>C = No	I^1 = TA<br>I^2 = TA<br>C = TA	Yes<br><br>No dropout	5/9	1. Fear of Spiders questionnaire (6 items)<br><br>Baseline<br>Post-intervention (within 1 week of baseline)	1. I^1 : Yes<br>I^2 : Yes	Insufficient data to calculate effect sizes

					display (Division dVisor)<br>C = Virtual reality							
Rothbaum et al. 1995) [51]<br><br>USA<br><br>Acrophobia	N = 31 (Randomized)<br>N = 20, I = 12, C = 8) (Analyzed)<br>University students with symptoms of acrophobia<br>Recruitment: Not reported	M = 20<br>SD = 4<br>F = 40%	I = 7 x weekly sessions of virtual reality with railing and edge hardware to mimic height situations<br>C = Wait-list control	Indicated<br><br>(Participants indicated substantial fear and avoidance of heights according to the Acrophobia Questionnaire)	I = Virtual reality<br>C = N/A	I = No<br>C = N/A	I = TA<br>C = N/A	No	5/9	1. AQ<br><br>Baseline<br>8 weeks	8 weeks:<br>Yes	8 weeks:<br>2.66
Vansteenwegen et al. (2007) [52]<br><br>BEL<br><br>Spider phobia	N = 54, I^1 =18, I^2 = 18, C = 18 (Randomized)<br>N = 52, I^1 = 18, I^2 = 17, C = 17 (Analyzed)<br>Student volunteers<br>Recruitment: NR	M = 19.2<br>SD = NR<br>F = 96.3%	I^1 = Exposure therapy video (11 x 60 seconds + black screen of 60s between each video) of a tarantula spider in a single location<br>I^2 = Exposure therapy video (11 x 60 seconds + black screen of 60s between each video) of a tarantula spider in 3 different locations<br>C = 11 x 60 second video presentation of location without spider.<br><br>Intervention conditions were approximately 30 minutes (estimate).	Treatment<br><br>SPQ ≥ 16 (criterion corresponds with lowerbound of the 95% reliability interval of the mean of comparable group of 45 Dutch female spider phobics seeking treatment (Muris & Merckelbach, 1996))	I^1 = Video<br>I^2 = Video<br>C = Video	I^1 = No<br>I^2 = No<br>C = No	I^1 = PSH<br>I^2 = PSH<br>C = PSH	No	3/9	1. Fear rating (11 point scale)<br><br>Baseline<br>Post intervention (~30 minutes)	Post-intervention<br>1. I^1 : Yes<br>I^2 : Yes	Post-intervention:<br>1. I^1 : 1.35<br>I^2 : 1.35
**Stress (n = 2)**												
Chiauzzi et al. (2008) [53]	N = 240, I^1 = 80, I^2 = 80, C = 80<br>College students	M = NR (18-24 yrs)<br>SD = NR	I^1 = Website containing standard text-based college health	Selective<br><br>(PSS>14)	I^1 = Internet<br>I^2 = Internet<br>C = N/A	I^1 = Yes<br>I^2 = Yes<br>C = N/A	I = PSH<br>C = N/A	Yes<br><br>Mixed	4/9	1. PSS<br><br>Baseline	1 month:<br>1. I^1 : No<br>I^2 : No	Insufficient data to calculate

 USA<br><br>Stress	with elevated levels of stress<br>Recruitment: Research coordinators set up tables in public areas on campus (e.g., student centers, cafeterias) advertising a "college health study"	F = 51.5% (of those reporting gender, N = 139)	information (no images).<br>I^2 = Website containing motivational feedback on completion of 5 online questionnaires: 1) physical stress, 2) life events, 3) daily hassles, 4) coping styles, 5) mood/depression) + tailored articles, strategies and interactive relaxation tools + weekly updates of peers' stories, ask the expert questions, and an 'emergency area' to help students deal with crises.<br><br>Intervention participants were instructed to visit their assigned website at least 4 times (minimum 20 minutes) over a 2 week period.<br><br>C = No intervention					model analysis		1 month<br>3 months<br>6 months	3 months:<br>1. I^1 : No<br>I^2 : No<br>6 months:<br>1. I^1 : No<br>I^2 : No	effect sizes
Plante et al. (2003) [54]<br><br>USA<br><br>Stress	N = 154<br>Introductory psychology students<br>Recruitment: volunteered to participate to fulfill requirements for undergraduate psychology class	M = 19<br>SD = Not reported<br>F = 66.2%	I^1 = 1 x 20 min session of a brisk outdoor walk around campus<br>I^2 = 1 x 20 min session of virtual reality helmet designed to simulate same walk as I^1 while walking on treadmill<br>I^3 = 1 x 20 min session of virtual reality helmet alone without walking on treadmill<br>C = 1 x 20min session of walking on treadmill	Universal	I^1 = N/A<br>I^2 = Virtual reality<br>I^3 = Virtual reality<br>C = None	I^1 = No<br>I^2 = No<br>I^3 = No<br>C = No	I^1 = PSH<br>I^2 = PSH<br>I^3 = PSH<br>C = PSH	Yes<br><br>No dropout	6/9	1. AD-ACL – Energy<br>2. AD-ACL – Tiredness<br>3. AD-ACL – Tension<br>4. AD-ACL - Calmness<br><br>Baseline<br>Immediately post-intervention (~20 mins)	1. I^1, I^2, C > I^3<br><br>Overall sample data for outcomes 2, 3 and 4 not reported (categorized by gender)	Insufficient data to calculate effect sizes
**Social anxiety (n = 1)**												
Botella et al. (2010) [55]<br><br>ESP	N = 127, I^1 = 62, I^2 = 36, C = 29<br>Students with social phobia<br>Recruitment:	M = 24.4<br>SD = 5.78<br>F = 79.2%	I^1 = 2 months to complete "Talk to me": online CBT and exposure for fear of public speaking	Treatment<br><br>Met DSM-IV criteria for social	I^1 = Internet<br>I^2 = N/A<br>C = N/A	I^1 = Yes<br>I^2 = No<br>C = N/A	I^1 = SA<br>I^2 = TA<br>C = N/A	Yes<br><br>LOCF	5/9	1. Fear (TB)<br>2. Avoid (TB)<br>3. Beliefs (TB)<br><br>Baseline	2 months:<br>1. I^1 : Yes<br>I^2 : Yes<br>2. I^1 : Yes<br>I^2 : Yes	2 months:<br>1. I^1 : 1.83<br>I^2 : 1.88<br>2. I^1 : 2.09<br>I^2 : 1.85

Fear of public speaking (social anxiety)	Advertisements on university campus		I^2 = 2 sessions per week for 2 months therapist-delivered CBT and exposure for fear of public speaking<br>C = Wait-list control	phobia						2 months<br>12 months	3. I^1 : Yes<br>I^2 : Yes<br>12 months:<br>1. I^1 : NR<br>I^2 : NR<br>2. I^1 : NR<br>I^2 : NR<br>3. I^1 : NR<br>I^2 : NR	3. I^1 : 1.83<br>I^2 : 1.95<br>12 months:<br>Insufficient data to calculate effect sizes
**Computer-related anxiety (n = 1)**												
Farnill (1985) [56]<br><br>AUS<br><br>Computer-related anxiety	N = 21, I^1 = 7, I^2 = 7, C = 7<br>(Randomized)<br>Analyzed sample size not reported<br>Female undergraduate students enrolled in introductory psychology classes at a college in Victoria, Australia.<br>Recruitment: Students voluntarily participated.	M = 22.3<br>SD = 3.7<br>F = 100%	I^1 = 15 minutes of hypnosis treatment including induction & deepening procedures, positive suggestion about computers & computing + visualization techniques.<br>I^2 = 20-30 minutes of computer-assisted biofeedback via either EMG feedback of frontalis muscle activity (computer graphics were aiming to land a plane) or peripheral temperature feedback from index finger (controlling level of bulb thermometer). C = Wait-list control	Universal	I^1 = None<br>I^2 = Computer program<br>C = N/A	I^1 = No<br>I^2 = No<br>C = N/A	I^1 = TA<br>I^2 = SA<br>C = N/A	NR	4/9	1. STAI (state)<br><br>Baseline<br>7-10 days	Post-intervention:<br>1. I^1 : Yes<br>I^2 : Yes	Insufficient data to calculate effect sizes
**Posttraumatic stress (n = 1)**												
Lange et al. (2001) [57]<br><br>NLD<br><br>Post-traumatic stress & grief	N = 30, I = 15, C = 15<br>(Randomized)<br>N = 25, I = 13, C = 12<br>(Analyzed)<br>Students who had experienced a traumatic event in at least the previous 3 months<br>Recruitment: recruited from a	M = 22<br>SD = 4.9<br>F = 64%	I = 2 x 45 minute sessions per week for 5 weeks of "Interapy": Internet-mediated communication with therapist concerning structured writing assignments<br>C = Wait-list control	Selective<br><br>Experience of a traumatic event in at least the previous 3 months	I = Internet<br>C = N/A	I = Yes<br>C = N/A	I = MC<br>C = N/A	No	6/9	1. IES - intrusions<br>2. IES -avoidance<br><br>Baseline<br>5 weeks<br>6 weeks post-intervention	5 weeks:<br>1. Yes<br>2. Yes<br>6 weeks post-intervention:<br>No control data at follow-up	5 weeks:<br>1. 0.52<br>2. 1.10<br>6 weeks post-intervention:<br>No control data at follow-up

	pool of 500 students in return for course credit											
**Generalized anxiety disorder (Academic worry) (n = 1)**												
Wolitzky-Taylor & Telch (2010) [58]<br><br>USA<br><br>Generalized anxiety disorder (Academic worry)	N = 113, I^1 = 33, I^2 = 33 , I^3 = 29, C = 18<br>College students from large university who experience clinically significant worry<br>Recruitment: Email and verbal announcement to wide range of academic departments on the researchers' laboratory website, flyers at the university counseling centre and academic enrichment & mentoring programs, and other academically oriented organizations	85% undergraduates<br><br>M = NR<br>SD = NR<br>F = 75.2%	I^1 = 1-month Worry Exposure including training session (35-45 mins), and worry image loop tape (3 x 20-30 min sessions/wk)<br>I^2 = 1-month Expressive Writing online (3 x 20 min sessions/wk) including training session (35-45mins)<br>I^3 = 1-month Pulsed Auto-Photic Stimulation (APS) on iPod sized control board with headphones (3 x 35 min sessions per week).<br>C = Wait-list control<br><br>All interventions included filling out online home practice adherence logs after each session	Indicated<br><br>Clinically significant worry on AWQ (i.e., moderate distress and/or interference due to academic worry).	I^1 = Internet + Loop tape<br>I^2 = Internet<br>I^3 = Internet + APS<br>C = N/A	I^1 = Yes<br>I^2 = Yes<br>I^3 = Yes<br>C = N/A	I^1 = PSH<br>I^2 = PSH<br>I^3 = PSH<br>C = N/A	Yes<br><br>MLE	5/9	1. AWQ<br>2. PSWQ<br><br>Baseline<br>1 month<br>3 months	1 month:<br>1. I^1 : Yes<br>I^2 : No<br>I^3 : Yes<br>2. I^1 : Yes<br>I^2 : No<br>I^3 : Yes<br>3 months:<br>No control data at follow-up	1 month:<br>1. I^1 : 0.41<br>I^2 : 0.07<br>I^3 : 0.87<br>2. I^1 : 0.54<br>I^2 : 0.44<br>I^3 : 0.81<br>3 months:<br>No control data at follow-up

Notes: SA = self-administered therapy, PSH = predominantly self-help, MC = minimal-contact therapy, TA = predominantly therapist administered treatments, MLE = Maximum Likelihood Estimation, ED = Eating disorder, BMI = Body Mass Index, PB = Post baseline, PI = Post-intervention, ID = Insufficient data, NR = Not reported, LOCF = Last observation carried forward, GLMM = General linear mixed models, MLE = Maximum likelihood estimation.

Measures: BAI = Beck Anxiety Inventory, BDI = Beck Depression Inventory, DASS = Depression, Anxiety and Stress Scales, STAI = State Trait Anxiety Inventory, K-10 = Kessler Psychological Distress Scale, Fear (TB) = Target behavior (fear of social situations), Avoid (TB) = Target behavior (avoidance of social situations), Belief (TB) = Target behavior (belief in catastrophic thoughts regarding social situations), TAI = Test Anxiety Inventory, PSS = Perceived Stress Scale, ASI = Anxiety Sensitivity Index, IES = Impact of Events Scale, AD-ACL = Momentary Moods States Checklist, AQ = Acrophobia Questionnaire, AWQ = Academic Worry Questionnaire, PSWQ = Penn State Worry Questionnaire, CORE-OM = Clinical Outcomes in Routine Evaluation – Outcome Measure.

Study<br>Country<br>Disorder or symptoms targeted	Participants<br>(# randomized)<br>(# analyzed, if not ITT)	Age<br>(M, SD)<br>Sex (%F)	Intervention description	Type	Technology used	Distal?	Human contact?	ITT?	Quality rating	Primary outcome/<br>Measurement points	Significant interaction between time and group, favoring intervention group?	Effect size between intervention and the control (Hedge's *g* unless otherwise specified)
**Psychological distress (n = 1)**												
Freeman et al. (2008) [59]<br><br>GBR<br><br>Psycho-logical distress	N = 283, I^1 = 141, I^1 = 142 (Randomized)<br>Analyzed sample size not reported<br>Undergraduate & graduate students who felt "stressed out" or "low"<br>Recruitment: All students at university invited by email to participate in a study for students who were "stressed out or feeling low"	Median = 21<br>Range = 18-56<br>SD = NR<br>F = 70.0%	C = Information about common student problems only.<br>I^2 = Information about common student problems + online mutual support group via electronic bulletin board.<br><br>Both interventions were delivered over 10 weeks.	Selective<br><br>Self-reported low mood or stress	C = Internet<br>I^2 = Internet	C = Yes<br>I^2 = Yes	C = SA<br>I^2 = SA	No	5/9	1. CORE-OM<br><br>Baseline<br>10 weeks	1. No (I = C)	Insufficient data to calculate effect sizes
**Hardiness and acculturation (n = 1)**												
Kanekar et al. (2009) [60]<br><br>USA<br><br>Hardi-ness and accultur-ation	N = 60, I = 30, C = 30 (Randomized)<br>N = 39 (Analyzed)<br>Indian Asian International students<br>Recruitment: E-mails and newsletters	M = 24.67<br>SD = 2.68<br>F = 12.8%	I = 3 sessions over 2 months, online instruction to promote social support, hardiness and acculturation + fortnightly e-mail reminders<br>C = 3 sessions over 2 months, online instruction focused on general wellbeing (eating, physical activity, positive thinking)	Universal	I = Internet<br>C = Internet	I = Yes<br>C = N/A	I = SA<br>C = N/A	No	3/9	1. K-10<br><br>Baseline<br>2 Months	2 months:<br>1. No (I = C)<br><br>Both groups showed significant within-group improvement	Insufficient data to calculate effect sizes
**Internet addiction (n = 1)**												
Su et al. (2011) [61]<br><br>CHN<br><br>Internet	N = 65, I^1 = 17, I^2 = 16, I^3 = 16, C = 16 (Randomized)<br>N = 59, I^1 = 17, I^2 = 12, I^3 = 14, C = 16 (Analyzed)	M = 22.31<br>SD = 2.62<br>F = 69.2%	I^1 = 1 x 30 min session of "Healthy Online Self-Help Centre": motivational interviewing and client centered approach, 4	Indicated<br><br>Internet addiction (≥5 YDQ score) or	I^1 = Internet<br>I^2 = Internet<br>I^3 = Internet<br>C = N/A	I^1 = No<br>I^2 = Yes<br>I^3 = No<br>C = N/A	I^1 = SA<br>I^2 = SA<br>I^3 = SA<br>C = N/A	No	6/9	1. Hours online per week<br><br>Baseline<br>1 month PB	Post-intervention:<br>1. All intervention groups showed significant within-group improvement.	Insufficient data to calculate effect sizes

addiction	Undergraduate and graduate students with Internet addiction or high risk Internet dependence<br>Recruitment: Posters distributed on campus		modules, assessment, feedback, goal setting, cognitive restructuring. Conducted in laboratory setting.<br>I^2 = 1 x 30 min session of “Healthy Online Self-Help Centre”: Conducted in own setting<br>I^3 = 1 x 15 min session of “Healthy Online Self-Help Centre”: Conducted in laboratory setting with no tailored feedback/ interactivity<br>C = No intervention	high risk Internet dependence (3-4 on YDQ) and being online >14 hours per week							The control group did not show significant within-group improvement.<br><br>Insufficient data to determine effectiveness of each intervention relative to the control or calculate effect sizes	

Notes: SA = self-administered therapy, PSH = predominantly self-help, MC = minimal-contact therapy, TA = predominantly therapist administered treatments, MLE = Maximum Likelihood Estimation, ED = Eating disorder, BMI = Body Mass Index, PB = Post baseline, PI = Post-intervention, ID = Insufficient data, NR = Not reported, LOCF = Last observation carried forward, GLMM = General linear mixed models, MLE = Maximum likelihood estimation.

Measures: BAI = Beck Anxiety Inventory, BDI = Beck Depression Inventory, DASS = Depression, Anxiety and Stress Scales, STAI = State Trait Anxiety Inventory, K-10 = Kessler Psychological Distress Scale, Fear (TB) = Target behavior (fear of social situations), Avoid (TB) = Target behavior (avoidance of social situations), Belief (TB) = Target behavior (belief in catastrophic thoughts regarding social situations), TAI = Test Anxiety Inventory, PSS = Perceived Stress Scale, ASI = Anxiety Sensitivity Index, IES = Impact of Events Scale, AD-ACL = Momentary Moods States Checklist, AQ = Acrophobia Questionnaire, AWQ = Academic Worry Questionnaire, PSWQ = Penn State Worry Questionnaire, CORE-OM = Clinical Outcomes in Routine Evaluation – Outcome Measure.
